# Isolated Axillary Lymphadenitis Due to Bartonella Infection in an Immunocompromised Patient

**DOI:** 10.7759/cureus.5456

**Published:** 2019-08-21

**Authors:** Arjun Balakumar, Belinda Lao, Dimitrios Papanagnou, Xiao Chi Zhang

**Affiliations:** 1 Emergency Medicine, Sidney Kimmel Medical College at Thomas Jefferson University, Philadelphia, USA; 2 Emergency Medicine, Thomas Jefferson University, Philadlephia, USA; 3 Emergency Medicine, Thomas Jefferson University, Philadelphia, USA

**Keywords:** bartonella henselae, adult, cat scratch disease, clinical images, outcome, treatment

## Abstract

Bartonella henselae is a relatively uncommon pathogen that can present as a serious disease in immunocompromised patients. We present a case of a 76-year-old man with stable chronic lymphocytic leukemia (CLL) who presented to the emergency department (ED) with an onset of right axillary lymphadenitis after recovering from a recent cat bite on the ipsilateral finger. Doppler ultrasound demonstrated an irregular, circumscribed 5cm x 4cm, hypoechoic mass with mild vascular flow consistent with an enlarged abnormal lymph node. The patient was diagnosed with cat scratch disease and discharged on oral antibiotics with spontaneous drainage of the purulent materials in subsequent outpatient oncology visits. This case highlights the classic presentation of this rare disease in an immunocompromised patient with feline contact. Early antibiotics should be considered for at-risk and immunocompromised patients due to low sensitivity and specificity for Bartonella serologic tests. CLL can also present with similar progressive lymphadenopathy with severe systemic symptoms and extranodal involvement that requires emergent oncologic interventions and diagnostic vigilance.

## Introduction

Bartonella henselae (B. henselae) is an uncommon, intracellular, gram-negative bacterium that tends to affect younger or immunocompromised patients in the setting of cat exposure. Greater than 22,000 diagnosed cases of cat scratch disease (CSD) are made annually each year, with an estimated 2000 hospitalizations [1]. Untreated disseminated Bartonella infection can result in bacteremia, encephalopathy, endocarditis, meningitis and death [2, 3].

## Case presentation

A 76-year-old man presented to the emergency department (ED) with a sudden onset of a tender swollen right axillary gland. His cat bit him on the ipsilateral right middle finger two weeks ago, resulting in transient but self-resolved localized swelling and suppuration. One day prior to his ED visit, he noticed a soft, rapidly progressing tender lump in the right axilla, with no other global lymphadenopathy. The patient’s past medical history was significant for chronic lymphocytic leukemia (CLL) in remission, chronic hepatitis B, chronic kidney disease, and dermatitis. Review of systems was negative for fevers, chills, weight loss, or night sweats. He denied any new travels or medications. 

**Figure 1:**
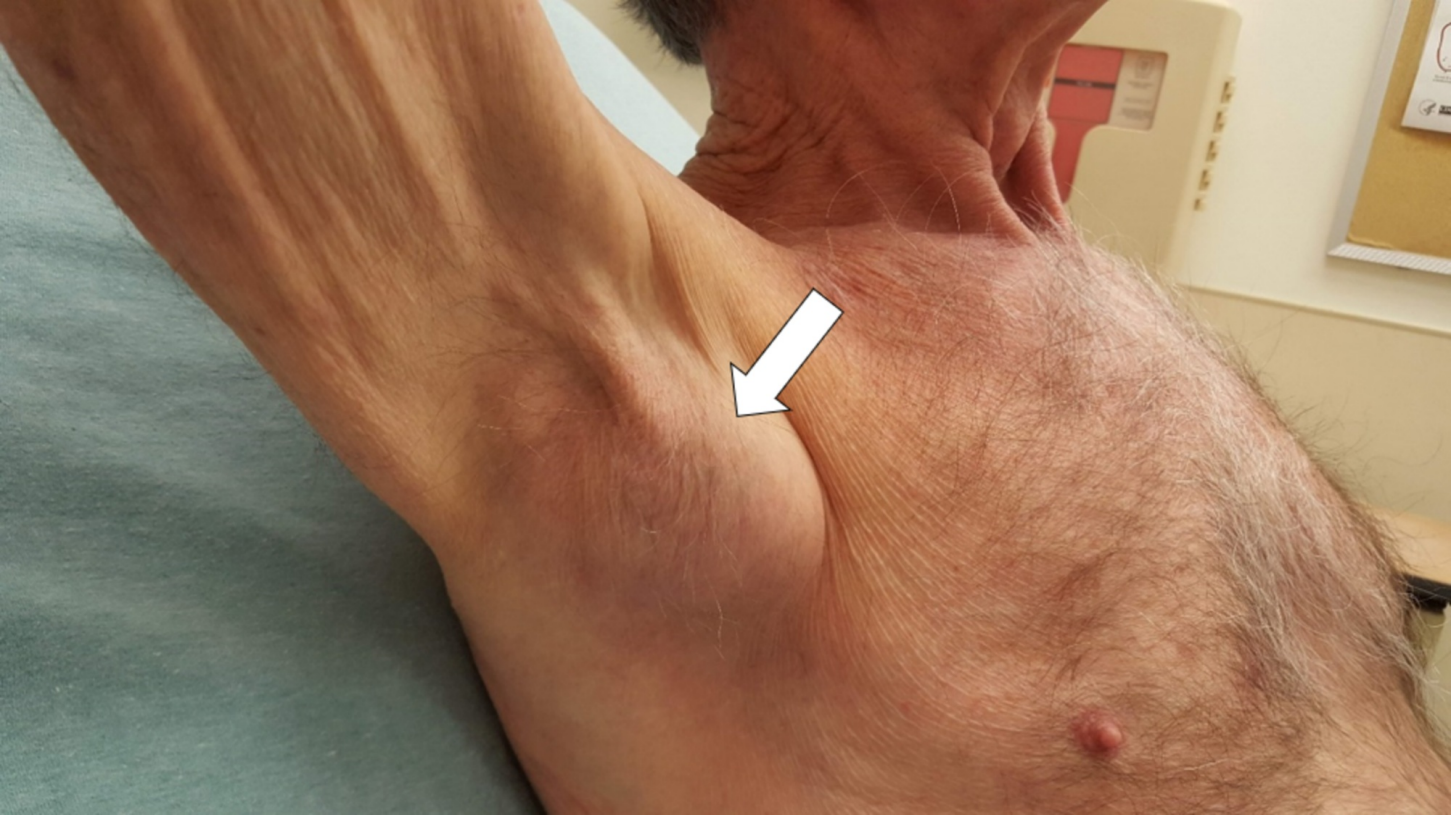
The patient has a tender right axillary lymphadenopathy (white arrow) after a cat bite on the ipsilateral finger 2.5 weeks prior to arrival.

On ED arrival, the patient’s vital signs were: blood pressure 140/60 mmHg; heart rate 72 beats/min; respiratory rate 18 breaths/min; temperature 36.4^o^C (97.5^o^F); and SaO_2_ 96% (room air). His lymphatic exam was significant for an uncomfortable 5cm x 4cm mobile mass in the right axilla without fluctuance or erythema (Figure 1). He had two small healing puncture wounds on his right long distal fingertip without infection, swelling or lymphangitis. The remainder of his exam was unremarkable.

**Figure 2:**
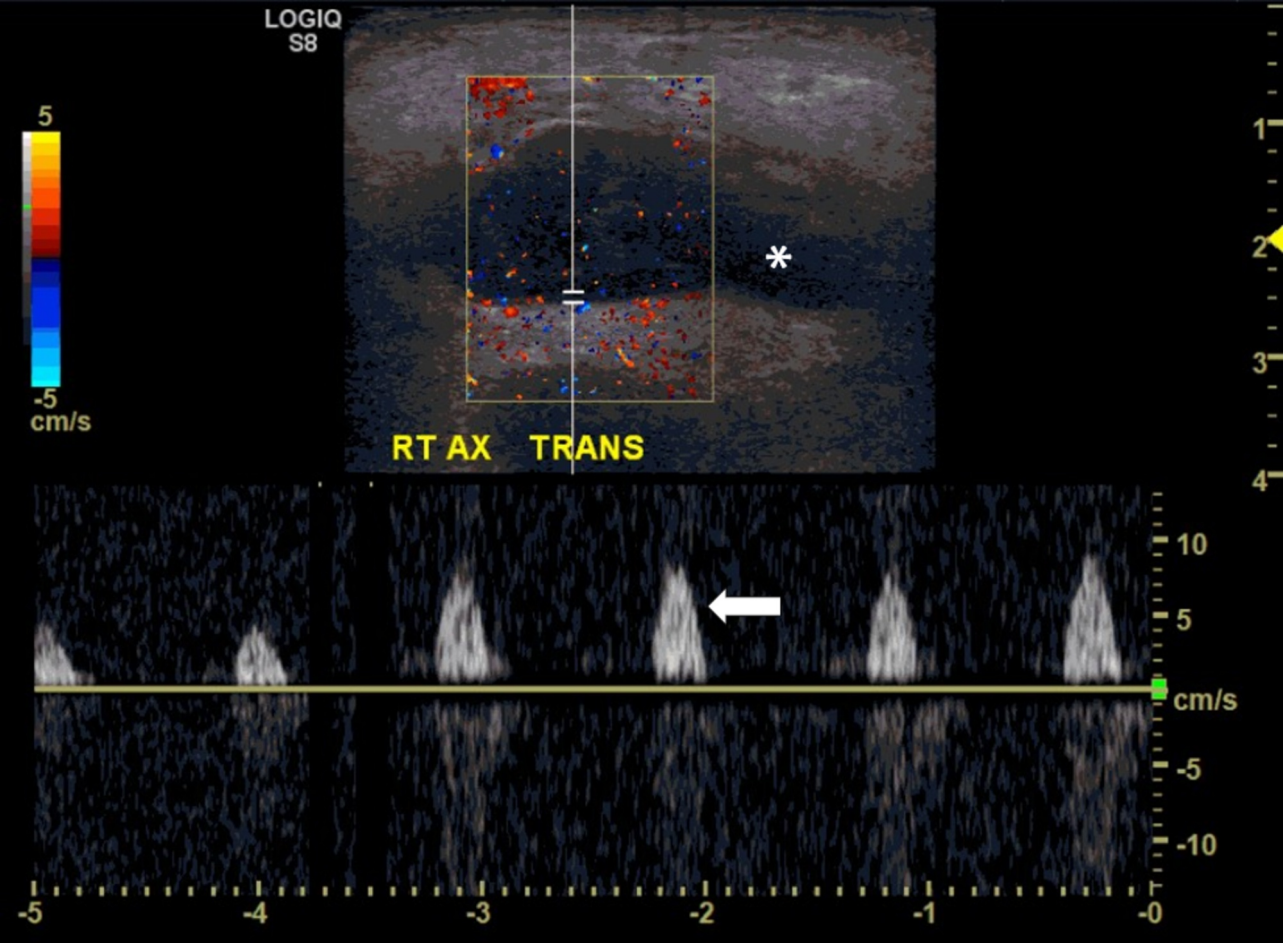
There is a 4.4cm x 3.3cm x 1.6cm, irregular, circumscribed hypoechoic mass (marked with *) with the mild vascular flow (white arrow) within the subcutaneous fat of the right axilla suggestive of an enlarged abnormal lymph node.

Initial laboratory results were significant for a white blood cell count of 10.2 x 10^3^/uL (reference range: 4.0-11.0 x 10^3^ /uL) with 42% lymphocytes (reference range: 20-44%) and minimally elevated urate level of 6.2 mg/dL (reference range: 2.5-6mg/dL). A Doppler ultrasound revealed a 4.4cm x 3.3cm x 1.6cm, irregular, circumscribed hypoechoic mass with the mild vascular flow within the right axilla subcutaneous fat suggestive of an enlarged abnormal lymph node (Figure 2). 

The patient was diagnosed with CSD and discharged with azithromycin and a close outpatient oncology follow-up for biopsy. Three weeks later, his axillary lymph node spontaneously ruptured with purulent drainage and symptom-relief. He did not pursue further biopsy. His final blood cultures and serologic titers for Bartonella (immunoglobulin M [IgM] and immunoglobulin G [IgG]) were negative.

## Discussion

This case highlights the classic presentation of CSD in an immunocompromised patient with feline contact. B. henselae is the primary pathogen in CSD and is frequently transmitted to humans through asymptomatic juvenile cat scratches or bites, and less frequently through dogs and fleas, CSD is often diagnosed in young men, with peak incidence during cooler and humid climates in the US [4-6].

CSD initially presents as a solitary lesion at the inoculation site, then develops into vesicular, erythematous, and papular phases within 3-10 days. Less common manifestations include maculopapular eruptions, erythema nodosum, and thrombocytopenic purpura [7]. Axillary and epitrochlear lymphadenopathy may develop weeks later. The nodes range from 1-5 cm and can be tender with overlying skin erythema and suppurate, but most resolve between one to four months [8]. CSD can have visceral involvement such as intra-abdominal granulomas with symptoms including fever, pain, weight loss, and hepatosplenomegaly [9]. Additional manifestations include Parinaud’s oculoglandular syndrome (2-8%) and neuroretinitis (1-2%) [10]. Parinaud’s oculoglandular syndrome refers to the constellation of symptoms of ocular involvement including regional lymph node involvement, as well as direct infection of the conjunctiva and eyelids. Symptoms include foreign body sensation and copious discharge, but serious long-term sequelae are not common [11]. Neuroretinitis and ensuing vision loss secondary to optic nerve edema may occur in up to 1-2% of patients. Involvement is largely unilateral, and signs include retinal “cotton-wool spots” and macular exudates (“macular star”) [12]. Musculoskeletal, pleural, and deep neck involvement are rare but can represent serious manifestations [11]. Disseminated B. henselae can present in immunocompromised patients as widespread violaceous cutaneous papules and visceral involvements [13]. The gold standard test is IgM or IgG immunofluorescence with sensitivity ranging from 20-90% [14]. While PCR and skin testing are available, these tests are associated with even lower sensitivity (20-76%) [15]. Most patients with CSD do not have B. hensalae isolated on cultures. 

Treatment of Bartonella infection includes macrolides, aminoglycosides, and doxycycline. Alternative regimens may include rifampin or trimethoprim-sulfamethoxazole. Most immunocompetent patients require little more than supportive care; however, antibiotics are recommended for immunosuppressed patients to prevent more serious disease sequelae; rifampin can be added as an adjunctive for severe lymphadenopathy, or ocular or hepatosplenic involvement [15].

Our case illustrates the importance of treating Bartonellainfection in an immunosuppressed patient despite a negative serologic workup. Similar to herpes simplex, where the culture and PCR sensitivity is 50% and 80% respectively, Bartonella* *infection* *should be diagnosed and treated based on exam findings and high clinical suspicion [16]. This case was further complicated by CLL, which can present dangerously as Richter syndrome, occurring approximately 2-10% of CLL patients during their disease process, with aggressive lymphadenopathy (64%), systemic symptoms (59%), and extranodal involvement (41%) [17-19]. Additional non-oncologic diagnoses for lymphadenopathy include resolving cellulitis and tularemia, however, they are less likely in this case due to lack of outdoor exposure or dermal infection on initial presentation [20].

## Conclusions

CSD is a rare, but classic manifestation of Bartonellainfection for patients presenting with new-onset lymphadenopathy with feline exposure. Immunosuppressed patients with high clinical suspicion for CSD should receive antibiotics due to low sensitivity and specificity of the serologic testing. Patients with CLL can also present with similar, but severe progressive lymphadenopathy requiring diagnostic vigilance and clinician acumen.
